# General practitioners’ perspectives on discontinuation of long-term antidepressants in nursing homes

**DOI:** 10.1080/13814788.2022.2038131

**Published:** 2022-03-30

**Authors:** Ellen Van Leeuwen, Sibyl Anthierens, Mieke L. van Driel, An I. M. De Sutter, Evelien van den Branden, Thierry Christiaens

**Affiliations:** a Clinical Pharmacology Unit, Department of Basic and Applied Medical Sciences, Ghent University, Gent, Belgium; b Unit General Practice Ghent University, Department of Public Health and Primary Care, Ghent University, Gent, Belgium; c Family Medicine and Population Health, University of Antwerp, Antwerp, Belgium; d Primary Care Clinical Unit, Faculty of Medicine, The University of Queensland, Brisbane, Australia

**Keywords:** Discontinuation, deprescribing, long-term antidepressant use, nursing homes, general practice, qualitative research, depression

## Abstract

**Background:**

Long-term use of antidepressant drugs (AD), much longer than recommended by guidelines, in nursing homes (NH) is common. NH home residents may have a relatively higher risk of adverse events. Moreover, in an NH setting nursing staff and relatives are intensively involved in the decision-making process. In many countries, General Practitioners’ (GPs) provide care for residents in NHs. Little is known about GPs’ perspectives on discontinuation of long-term AD in NH residents.

**Objectives:**

To explore GPs’ views of discontinuing long-term AD in NH residents.

**Methods:**

An exploratory qualitative study, with semi-structured interviews, was conducted with a purposive sample of 20 Belgian GPs. Interviews, conducted over six months in 2019, were analysed by thematic analysis.

**Results:**

Twenty interviews were conducted until data saturation. The first theme, ‘reluctance to rock the boat: not worth taking the risk’, describes that GPs perceive discontinuation as an unpredictable risk without clear benefits. GPs’ main concern was the risk of destabilising the fragile balance in an older patient. Second, ‘it takes at least three to tango’, captures the unspoken alliance between GPs, nursing staff and relatives and suggests that agreement of at least these three partners is required. The third, ‘Opening the door: triggers to discontinue’, points to severe health problems and dementia as strong facilitators for discontinuation.

**Conclusion:**

Discontinuation of long-term AD in NHs is a complex process for GPs. More evidence and attention to the role nursing staff and relatives play are needed to better guide the discontinuation of AD in older NH patients.

KEY MESSAGES

- Our study suggests that once an older person in a nursing home setting has prescribed an AD, GPs hesitate to discontinue the AD after the recommended duration.
- A greater focus on the nursing staff and relatives’ important role play may support GPs and NH residents when discontinuing long-term AD.

## Introduction

The use of antidepressants (AD) by older people in nursing homes (NH) is common. In the UK, 33.6% of NH residents take an SSRI daily and in the US the rate is even higher with 46% of older adults taking AD [1,2]. Rates are similar in Belgium (40%) and many other European countries [3,4]. Overall, nursing home residents receive more AD than non-institutionalised older people [1,5].

Evidence suggests that the effect of AD continuation in older adults after improvement is uncertain because the few available studies were not representative [6]. Overall, there is a lack of studies in older people, especially in frail older adults or the very oldest (80 years and older) [7]. AD is associated with a range of adverse events such as sleep disturbance, weight gain, and gastrointestinal problems [8], alongside feeling emotionally numb and becoming physically dependent [9,10]. In older people, there is an increased risk of falls and hip fractures [11,12], hyponatraemia, urinary incontinence and upper gastrointestinal bleeding [13]. Older people are also more at risk of adverse events and interactions because of altered pharmacokinetics and pharmacodynamics, comorbidities and polypharmacy.

Guidelines recommend that ADs should be continued for a year after remission of a single episode of depression in older adults and for at least two years in those with a high risk of relapse [14,15]. However, these guidelines are based on consensus and not on evidence from trials.

Considering these observations, long-term AD use, much longer than recommended by guidelines, without a clear indication to continue, e.g. in patients who have been stable and feeling well for many years while using an AD, seems relevant. Utilisation data of AD in NH, however [1–4], suggests that discontinuation of long-term AD often does not happen.

Discontinuing AD is not easy and successful discontinuation depends on both patient-related and physician-related factors [16]. A recent paper describes GPs’ perspectives on discontinuation in the community [17], however, little is known about their perspectives on the discontinuation of long-term AD in residents of NHs. One study exploring GPs views on the broader topic of treatment decisions regarding depression found that NH residents were kept on AD even if the GPs and nursing staff felt uncertain whether it was effective [18]. GP’ perspectives on discontinuation of AD are essential as in many countries, including Belgium, GPs provide care (and prescribe) for residents in NHs. Therefore, we aimed to explore the views of GPs working in nursing homes regarding discontinuation of long-term AD, much longer than recommended, in nursing home residents.

## Methods

### Study design and participant recruitment

A qualitative study was conducted with GPs recruited from two regions (Ghent, Dendermonde) in Belgium. Purposive sampling was used to ensure the sample represented a wide variation of personal characteristics such as practice type and grade of urbanisation. All GPs provided regular clinical care for residents living in different nursing homes. Invitations to participate in the study were sent by email to university teaching networks and professional GP networks and followed up by two researchers (EVL, EVDB) by phone. All GPs provided informed written consent.

### Setting

Belgium has a well-established network of home care services that allow older people to live in their own homes as long as possible. Therefore, residents in NHs have rather a high dependency. Approximately 75% of residents are care-dependent and more than a third have dementia [5]. The regular GP can continue to see their patients in NHs but often another GP takes over due to practical barriers such as distance. Each GP can prescribe without any restrictions. Regular medication reviews are recommended but have not yet been largely implemented in Belgian NHs. A typical NH in Belgium has around 100 residents and is visited by a median of 30 GPs. All GPs in a specific NH are coordinated by a peer GP who is responsible for training and quality of care initiatives in the NH.

### Data collection

A qualitative researcher/GP (EVL) and a GP-trainee (EVDB) conducted semi-structured face-to-face interviews between May and December 2019 using an interview guide based on the literature (Box 1) [17–21]. In brief, participants were asked about their experiences with discontinuation of long-term AD (much longer than recommended by guidelines without a clear indication to continue), barriers and facilitators to discontinuation and differences between discontinuation in older adults in NHs compared to older people living in the community. Prompts followed open-ended questions to gather further detail. The interviews were audio-recorded, transcribed verbatim, and transcripts were then checked for accuracy and anonymised. The interviews were conducted in the GPs’ practices. Interviews continued until data saturation occurred.

### Data analysis

All transcripts were uploaded in NVIVO v12 and coded thematically by two authors (EVL and EVDB). Data were analysed using reflexive thematic analysis [22]. Three authors (EVL, EVDB, SA) independently read five interviews and made notes on key topics and potential themes to begin developing an initial framework. To ensure the trustworthiness of the findings, the analysis (coding, developing categories and themes) was then discussed in the team and for further interviews codes and categories were refined and defined at different stages iteratively by two authors (EVL and EVDB). The initial framework was used throughout the analysis of the interviews and further amended and adopted. The main themes were identified, discussed and agreed upon by the full study team.

Box 1

**Table:** 

Interview Guide Part 1: - Try to remember at least 1 patient for whom discontinuing long term antidepressant use (>1 year) was successful **1. What was your experience and what was the process?** - Try to remember at least 1 patient for whom discontinuing long term antidepressant use (>1 year) was NOT successful **2. What was your experience and what was the process?** *[‘Prompts’: why did you discontinue, who started the conversation, how did it go, what worked well, what didn’t work so well, what was the role of other health professionals, psychological and physical capabilities of the patient …]* Part 2: **3. Which factors and circumstances make antidepressants (AD) discontinuation possible, in your opinion? Why?** **4. Which factors and circumstances are barriers to AD discontinuation in your opinion? Why?** *[“Prompts: necessary knowledge, skills and attitude are needed, perception of depression and monitoring of AD psychological and physical capabilities of the patient, consequences and impact of discontinuation of AD, what was the role of other health professionals/family/friends, influence of practice related issues, influence of external factors …”]* **5. Which factors and circumstances make AD discontinuation in the nursing home different from AD discontinuation in adults living at home? Why?** **6. Which factors and circumstances make AD discontinuation in nursing homes possible in your opinion? Why?** **7. Which factors and circumstances are barriers to AD discontinuation in nursing homes, in your opinion? Why?** Part 3: **8. What support would you need to discontinue AD in more of your patients? Why?** *[prompts: knowledge and attitude (guidelines/e-learning, training), resources, external factors, role of other health professionals/others]*

## Results

### Participants

Of the 24 contacted GPs, 20 GPs with different profiles agreed to be interviewed (demographics in Table 1). Their ages ranged from 30 to 68 years, twelve GPs were female and five were GP-coordinator. All interviews lasted 25–60 min.

**Table 1.** Demographics of the 20 FPs.

Age (range in years, years)	
25–35	5
35–45	4
45–55	7
55–65	3
>65	1
Average	44.6
Sex	
Female	12
Male	8
Area	
Rural	10
Urban	10
Type of practice	
Solo	5
Duo	3
Group	10
Community health centre	2
Coordinating FP in nursing home	
Yes	5
No	15

Three main themes were identified: ‘reluctance to rock the boat: not worth taking the risk’, ‘it takes at least three to tango’, ‘opening the door: triggers to discontinue the AD’.

Our GPs reported that most ADs were initiated before residents entered the nursing home and they were reluctant to discontinue an AD that had been taken for many years.

Table 2 presents quotes illustrating each of the themes.

**Table 2.** Quotes illustrating each of the themes.

Reluctance to rock the boat: not worth taking the risk	
**The fear of making things worse**	
Risk of destabilizing the fragile balance of the older patient	*‘If I take away the AD they can get very emotional and feel like there’s no point in living anymore.’ (GP13, F, 53y)*
Living in a nursing home as a depressive enhancing situation	*‘I think we often too quickly jump to the conclusion that if you are old you are depressed. As a doctor and as a society, we seem to think it comes with life.’ (GP11, F, 58y)*
Benefit of the doubt	*‘I have had some people in the nursing home where I noticed they were feeling down, in that case I start an AD and feel they improve. But are they improving due to the medication or are they adjusting to life in the nursing home and are they happier because of that?’ (GP18, M, 31y)‘Some patients are tired of life, they don’t feel like doing anything. Some of them really insist on it in the hope of achieving something with [the AD]. In that case I stop all psychotropic medicines except the antidepressant; you never know if it still does something.” (GP9, M, 47y)*
Discontinuation perceived as a negative intervention	*‘Especially when they are already quite old I think, is it still worth the trouble to do something about it? My reasoning in those cases tends to be along the lines of; is there any harm in them taking it, should we really take this away from them after all these years, they don’t seem to have had issues with it, should I really be taking it away from them in the end?” (GP20,F,32y)*
**Limited alternatives to support discontinuation**	
Limited alternatives to support discontinuation	*‘That is something I try to discuss with the nurses: keep an eye on that person, talk with them a little more. But the issue in nursing homes is that there is no time to do anything other than the basic care.” (GP2, F, 35y)*
It takes at least three to tango	
Request from nursing staff and relatives to start AD or pressure to continue	*‘Those people are there, are bored, are at the end of their life and are complaining to their family about the food, the toilet visits that are delayed, not getting any visitors,… Some residents are very negative towards their family and then the family will ask “can’t you give them something to be happier”. That is a request we get a lot.’ (GP12, F, 50y)*
Importance of the opinion of the family and the nursing staff	*‘I haven’t explicitly asked residents, no. Are you happier, I don’t ask that. If the daughter has no issues, then everything is fine.’ (GP3, F, 58y)*
Time and energy consuming process to involve the family and nursing staff	*‘There are many people in the nursing home with dementia for whom the treatment could be stopped. Why am I not doing it? A lack of time I guess. You need to discuss it with the nurses and relatives before you can change anything in the medication. It takes a lot of energy and actually the resident doesn’t realise it, but the relatives, well most of them, keep an eye on it. You need to get them on board. Sometimes that is difficult, very difficult; sometimes it is not.’ (GP8, F, 42)*
Nursing staff and relatives as supporting partner in the discontinuation process	*‘When stopping I also ask the nursing staff to let me, as well as the family, know if they see any changes in the behaviour.’ (GP 6, M, 48)*
Opening the door: triggers to discontinue the AD	
**Medication review as an opportunity?**	
AD as a safe drug	*‘If somebody falls, they often break a hip. Then I focus more on benzos. I must say that I am happy if it is only escitalopram, yes. If it doesn’t help, it doesn’t hurt too much either. In contrast to a benzo, you know that those don’t do anything anymore after so many years.’ (GP 4, M, 32)*
Deprescribing of the AD is not a priority	*‘In any case with polypharmacy you try to stop there, there often is an AD too and in a lot of people it can be stopped but I also think that the more medication they take the more difficult it is to stop the AD. I would consider [stopping] a statin, something for the stomach, something for blood pressure and sometimes an antipsychotic, but certainly not the antidepressant, that is really the last thing.’ (GP 2, F, 35y)*
**Severe health problems as trigger for discontinuation**	
	*‘It works best when people are very ill. In that case I see things going wrong and wonder if all this medication is really necessary. In these cases the family is more likely to say “ok we’ll try it and we’ll see what happens”.’ (GP 10, M, 65y)*
**Dementia makes discontinuation easy**	
	*‘We do taper in those suffering from dementia, that is much easier, because you want to improve their quality of life and the less medication they need to take the better, and when you notice that their cognitive functioning is deteriorating, in those cases you do not see value in an AD anymore. This is maybe the easiest group as you do not get any resistance.’ (GP 8, F, 42y)*

### Theme 1. Reluctance to rock the boat: not worth taking the risk

*The fear of making things worse*. The GPs’ main concern was the risk of destabilising the fragile balance in an older patient and they would feel responsible for possible worsening of their depressive feelings: ‘*If I take away the AD they can get very emotional and feel like there's no point in living anymore.’ (GP13).*

Their own perception and assumptions reinforced this fear that living in an NH is a depression-enhancing situation. They associate living in an NH with sadness, grief, and the end of life. As one GP explained: ‘*I think we often too quickly jump to the conclusion that if you are old you are depressed.’ (GP11).* Several GPs said that by continuing the AD they empathise with the depressing situation of living in an NH. ADs were perceived as the only suitable solution to coping with the situation patients were in.

The view that AD can help patients deal with their changed living conditions was also echoed in their thoughts on the initiation of an AD. They described that many residents felt sad after entering an NH. GPs believed that the AD helped patients feel less sad and supported them in accepting their new situation. Therefore, some GPs said they prescribed an AD for residents who were ‘tired of life’, as a way of enhancing their ‘lust for life’, whereas other GPs thought it was helpful for loneliness.

However, several GPs pointed out they had doubts about the effectiveness of the AD. They thought that improvement in mood and quality of life can also be achieved without the AD once the resident has adapted to the situation in the NH: ‘*I noticed they were feeling down, in that case I start an AD and feel they improve. But are they improving due to the medication or are they adjusting to life in the nursing home and are they happier because of that?’ (GP18).* Furthermore, GPs said it was difficult to evaluate the effect of the AD in older people with cognitive impairment as they may be unable to report symptoms or describe how they feel, and therefore they relied on observations of NH staff and relatives. Overall, although some GPs question the effectiveness of ADs, they give them the ‘benefit of the doubt’ and tend to emphasise in the positive effects: ‘*Some patients are tired of life, I stop all psychotropic medicines except the antidepressant, you never know if it still does something.’ (GP9).*

GPs considered AD safe in older people and were unaware of potentially harmful effects. Overall, they saw discontinuation as a negative intervention associated with no benefits compared to continuation. They regarded discontinuation of long-term AD as ‘taking something away’ from their patient and thought it was not worth taking the risk of discontinuation at the end of life. One GP explained: *‘they don’t seem to have had issues with it, should I really be taking it away from them in the end?’ (GP20).*

Reviewing the patient’s medication was perceived as time and energy-consuming. Our GPs reported that repeat prescribing of ADs is very common, it is the easiest option. GPs blame their own habits of repeat prescribing, but they did not show any inclination to change these routines. Furthermore, GPs found changing their routines and implementing a regular proactive review of the AD difficult. This would entail abandoning the comfortable path of maintaining the status quo. Indeed, some GPs describe themselves as ‘reactive’ and do not review proactively.

### Limited alternatives to support discontinuation

Several GPs mentioned they needed an alternative for the AD when they discontinued ensuring the patient remained stable. Additional support was considered essential in the process of discontinuation. One GP explained: ‘*there is, in my opinion, not enough psychological counselling, so what can we fall back on when we stop the AD?’ (GP12)*

The availability of psychological support in an NH is often limited. Some GPs thought that an older patient would resist or be unable to participate in psychotherapy. Therefore, different approaches were used to reduce the risk of worsening after discontinuation. For example, they ask nurses to provide a close follow-up throughout the withdrawal process and to spend more time talking to the patient during the day. The occupational therapist can involve the patient in organised activities and ask relatives to walk with their relatives. However, lack of time and the high workload of allied healthcare professionals limits the use of non-pharmacological approaches.: ‘*That is something I try to discuss with the nurses: keep an eye on that person, talk with them a little more. But the issue in nursing homes is that there is no time to do anything other than the basic care.’ (GP2).* Some GPs also mentioned the lack of specific guidelines for use of AD in NHs, in contrast with guidelines about antipsychotics.

### Theme 2. It takes at least three to tango

Our GPs described the decision to discontinue long-term AD as a complex process, especially in an NH. While decisions related to people living in the community depend on the perspectives of GPs and patients and their relationship, discontinuation in NHs also includes discussions with nursing staff and relatives.

GPs used the experience that patients, nursing staff or relatives never asked to stop the AD to justify continuing prescribing. Many mentioned they felt pressured by the nursing staff and relatives to continue and even anticipated resistance towards discontinuation as it was perceived to help the resident feel better. Moreover, a request for AD initiation often came from relatives as the elderly person had been sad or lonely: ‘*Those people are there, are bored, are at the end of their life and are complaining to their family about the food, the toilet visits that are delayed, not getting any visitors,… then the family will ask ‘can’t you give them something to be happier.’ That is a request we get a lot.’ (GP12).* This was an additional reason for GPs not to discontinue the long-term AD.

GPs mentioned that raising the issue of discontinuation could jeopardise their relationship with nursing staff or relatives and they did not want to take this risk. There was also a fear of being blamed or of guilt if the patient became more depressed after discontinuation. These issues were not actively discussed with the different stakeholders.

Indeed, GPs said they rarely had an open discussion about long-term AD discontinuation with the patient, because they assumed residents are less interested in their medicine use or because of the patient’s cognitive impairment. However, some thought that only a few patients would resist.

It seems that the fear of making the patient feel worse generated an unspoken alliance with the nursing staff and relatives, creating a shared goal not to disturb a frail equilibrium. This partnership is also illustrated in GPs’ respect of the nursing staff and relatives’ requests to continue the AD. GPs were sympathetic to the nursing staff’s concerns regarding discontinuation. Discontinuation requires observation and even more time and energy if patients are unstable after cessation, resulting in a higher workload. They realise that while for a physician it is easy to decide on discontinuation, the nursing staff caring for the patient 24/7 will have to pick up the pieces.

GPs reported that they rarely had a conversation about the AD with nursing staff or relatives. The resident’s AD was considered adequate if there were no complaints or questions from nursing staff and relatives based on their daily interactions: *‘I haven't explicitly asked residents, no. Are you happier, I don’t ask that. If the daughter has no issues, then everything is fine’ (GP3).* The importance GPs place on the opinions of nursing staff and relatives suggests that if long-term AD discontinuation is considered, agreement of at least three partners (GP, nursing staff and relatives) would be a prerequisite (i.e. it takes at least three to tango). Indeed, the GPs indicated that discussing the issue with the nursing staff and relatives and involving them in decision-making is essential: ‘*You need to get them on board’ (GP8).* However, they dread the time and energy this requires. As a result, others say they would not involve relatives if the patient agreed to discontinuation. However, discontinuation may be less likely: *‘If there are still relatives you need to include and convince them too. That is extra work. It has already happened that I start tapering the AD and the family calls me the same evening; why did you stop that?’ (GP2).*

However, in some situations, the alliance with the nursing staff and relatives can facilitate discontinuation as they can be an important partner in the discontinuation process (e.g. providing observations during discontinuation, spending more time with them as an alternative for the AD). One GP explained: *‘You can ask the family: take him home or to the cafe, go for a walk together, things that are helpful as support’* (GP6). Therefore, some GPs argued that it is worth their time to talk about discontinuation, believing the relatives can be persuaded to reconsider the AD and try discontinuation.

### Theme 3. Opening the door: triggers to discontinue

*Medication review as an opportunity?* GPs perceived a medication review as a major opportunity to reconsider the long-term AD and generate dialogue around the medication with other healthcare professionals (e.g. pharmacist, nursing staff …) and relatives and involved them in the decision-making process. Despite the additional workload, GPs found a face-to-face meeting useful for sharing opinions about the medicines and wellbeing of the patient. In particular, they valued the pharmacist’s pharmacological knowledge and the nursing staff’s daily observations. Other GPs expressed less enthusiasm and hesitated to participate in a time-consuming medication review.

A medication review is meant to weigh the benefits and risks of harm of each medication and reconsider potentially inappropriate medications focussing on the quality of life. However, GPs pointed out that compared with other drugs, long-term AD is often not a priority to change after a medication view: ‘*I think that the more medication they take, the more difficult it is to stop the AD. … that is the last thing.’ (GP2).* Even when reviewing medications after a fall, they considered long-term ADs as safe drugs that do no harm to the patient and are less likely to interfere with the quality of life: *‘If somebody falls, they often break a hip. Then I focus more on benzos. I must say that I am happy if it is only escitalopram, yes. If it doesn’t help, it doesn’t hurt too much either’ (GP4).*

Arrival in an NH was identified as a key moment for a medication review, certainly if there is also a change of GP. Some GPs thought it was easier to raise the issue of discontinuation in a new patient rather than a known patient. In contrast, others considered a new patient a barrier to discontinuation because of the time-demanding process of familiarising themselves with the patient and their psychosocial context. Overall, GPs found that knowledge of the initial depressive disorder, the triggers, and why an AD was initiated, facilitates discontinuation. They mentioned two specific health-related situations that are potent triggers to reconsider the AD and overrule their concerns about withdrawal symptoms or worsening sad feelings.

### Severe health problems as trigger for discontinuation

A severe acute illness was seen as a predictor of successful discontinuation. The patient’s poor health acts as a trigger to reconsider the benefits and risks of harms of long-term AD. When the patient’s quality of life deteriorates, GPs no longer consider the AD necessary to maintain quality of life. They said they did not need to convince the relatives in that situation. They accept the shift in treatment goals from prevention and cure to quality of life as a unique priority.

### Dementia makes discontinuation easy

All GPs were motivated to stop the AD in patients with cognitive impairment and dementia. In these patients, it was considered more important to minimise the number of drugs and AD was thought not to add much value: ‘*you want to improve their quality of life and the less medication they need to take the better’ (GP8).* They found it easy to initiate discontinuation because there is no resistance from the patient (who is not able to understand) and nurses and relatives agreed to discontinue once they had accepted that dementia was irreversible and the associated changed priorities. The GPs’ belief that ADs are ineffective in severe dementia was an additional argument to convince the relatives.

GPs agreed that severe dementia acts as a trigger to reconsider the AD, certainly if the AD was used to differentiate between early dementia and depression. However, they pointed out that if an AD is initiated for this indication, it is likely to be continued for years without reviewing. Nevertheless, a medication review acts as a strong facilitator to reconsider it and try discontinuation.

## Discussion

### Main findings

Our study unravels the complexities of GP decision-making in older persons in an NH who have been on long-term AD. This understanding is critical for improving medication safety in the elderly. Once an older person in a nursing home setting has been prescribed an AD, it is unlikely that GPs discontinue the long-term AD. Their main fear was the risk of destabilising the older patient’s fragile balance. We found that the nursing staff and relatives can be barriers and facilitators through their support during the discontinuation process. A medication review was perceived as an opportunity to reconsider medication. However, discontinuation of long-term AD was not a priority unless there were severe health problems or dementia. These are strong motivators to discontinue the AD. GPs also voiced a need for psychological support.

### Strengths and limitations

We purposely sampled 20 GPs from 2 different geographic regions in Belgium with different sexes, experiences, and backgrounds to ensure we fully captured a wide range of opinions and the data reached saturation. The level of care-dependence of elderly people living in an NH may vary between countries. Therefore, our findings may not reflect the experiences of GPs working in NH with less care-dependent residents. In this study, we focussed on the perspectives of GPs rather than those of nursing staff and relatives. These are also important to fully understand the dynamic and require further research.

### Comparison with existing literature

Our study confirms the barriers reported in previous studies investigating GPs’ views on discontinuing long-term AD in the community [17–21,23]. As in other studies, many of our GPs were not concerned about the risks associated with long-term use in adults and older adults [16,21,23]. They were much more concerned about the risk of relapse after discontinuation [17–21,23]. However, GPs’ concerns about relapse may be present before nursing home placement, influencing their reluctance to discontinue in NH. Data on the risks of relapse after discontinuation of long-term AD would be useful, however, a recent Cochrane review could not draw a firm conclusion about the discontinuation of long-term AD for depression in older adults living in the community or NH because of a lack of studies [24]. On the other hand, another Cochrane review could not conclude about continuing AD in older people in remission to prevent relapse or recurrence due to a lack of representative studies [6]. Our study identified time constraints, lack of support and limited availability of alternative treatments as other barriers reported elsewhere [17,19–21,23].

In the context of NH residents, a significant finding was the importance of the opinions of nursing staff and relatives, similar to previous qualitative studies investigating GPs’ views on deprescribing of medicines in general in NHs [25,26]. However, our study elicited the ambivalent role relatives and nursing staff play. GPs considered them allies, either supporting them against ‘rocking the boat’ (and continuing the AD) or as an important partner in seising an opportunity for discontinuation once they are convinced about the lack of effectiveness of AD. Our GPs suggested that relatives can play an active role in reducing the risk of relapse, which may help allay their fear of relapse. To our knowledge, this has not been reported before and illustrates the importance of involving caregivers such as nursing staff and relatives from the beginning of the discontinuation process.

As in the study by Iden [18], our GPs respond to a resident’s low mood by prescribing an AD. Sadness can be a core symptom of depression, but it is often a normal emotional response to the end-of-life process, accompanied by loss of functional ability and health and loss of friends and relatives [27]. When a resident is ‘sad’, the cause of sadness should be further explored and the possibility of a diagnosis other than depression should be ruled out, rather than reaching for ADs and underestimating the resident’s coping resources. Nursing staff could respond to this low mood by providing company and encouraging social contact with others, offering meaningful activities or supporting the resident’s own coping resources based on a person-centred approach [27,28]. Our GPs see continuing the AD as empathising with the depressing situation of living in an NH. Although an emphatic attitude to the patient’s situation is essential in caregivers, a person-centred approach supporting residents’ resources and coping strategies would be more appropriate [27–30]. Our study elicits a need to include relatives and nursing staff in these approaches as they have a major role in the care.

### Implications for practice and policy

Our GPs elicited the need to be educated on the lack of a clinically meaningful effect of long-term AD and the higher risk of AD-related adverse events in older persons. In line with this, and to provide clear guidelines for AD treatment duration in older adults, we need more studies investigating the effectiveness and safety of long-term AD, particularly in the oldest and most frail [24].

Second, the physician has a key role in discontinuation but the nursing staff and relatives are equally important. Therefore, it is essential that future discontinuation interventions also address the needs of nursing staff and relatives. Their active involvement in shared decisions to discontinue the long-term AD and in the discontinuation process needs to be encouraged.

Third, NH can facilitate the process by providing alternatives for the AD, such as psychosocial interventions and meaningful social activities tailored to the needs of the residents to reduce low mood. Implementing routine medication reviews can be useful but as long as discontinuation of long-term AD, much longer than recommended by guidelines, is perceived as a low or no priority, GPs’ motivation to discontinue AD will remain low. Considering medication reviews as a key solution to reduce unnecessary long-term AD treatment is overoptimistic.

## Conclusion

The decision to discontinue long-term AD in NH is a complex process for GPs and there are specific barriers. Most important is their alliance with nursing staff and relatives, which can be a significant opportunity as well as a barrier. Maintaining the status quo allays the fear of destabilising the resident’s fragile balance. However, questioning the need for AD can initiate a process that can improve an older person’s quality of life and mitigate the risks of polypharmacy. More attention to NH nursing staff and relatives as important partners in shared decision-making is required to reduce unnecessary long-term AD treatment.
